# Pericytes: The lung-forgotten cell type

**DOI:** 10.3389/fphys.2023.1150028

**Published:** 2023-03-23

**Authors:** Annelise T. Garrison, Rebecca E. Bignold, Xinhui Wu, Jill R. Johnson

**Affiliations:** ^1^ School of Biosciences, College of Health and Life Sciences, Aston University, Birmingham, United Kingdom; ^2^ Department of Molecular Pharmacology, Faculty of Science and Engineering, University of Groningen, Groningen, Netherlands; ^3^ Groningen Research Institute for Asthma and COPD, University Medical Centre Groningen, University of Groningen, Groningen, Netherlands

**Keywords:** pericyte, fibrosis, asthma, COPD, chronic obstructive pulmonary disease, pulmonary hypertension

## Abstract

Pericytes are a heterogeneous population of mesenchymal cells located on the abluminal surface of microvessels, where they provide structural and biochemical support. Pericytes have been implicated in numerous lung diseases including pulmonary arterial hypertension (PAH) and allergic asthma due to their ability to differentiate into scar-forming myofibroblasts, leading to collagen deposition and matrix remodelling and thus driving tissue fibrosis. Pericyte-extracellular matrix interactions as well as other biochemical cues play crucial roles in these processes. In this review, we give an overview of lung pericytes, the key pro-fibrotic mediators they interact with, and detail recent advances in preclinical studies on how pericytes are disrupted and contribute to lung diseases including PAH, allergic asthma, and chronic obstructive pulmonary disease (COPD). Several recent studies using mouse models of PAH have demonstrated that pericytes contribute to these pathological events; efforts are currently underway to mitigate pericyte dysfunction in PAH by targeting the TGF-β, CXCR7, and CXCR4 signalling pathways. In allergic asthma, the dissociation of pericytes from the endothelium of blood vessels and their migration towards inflamed areas of the airway contribute to the characteristic airway remodelling observed in allergic asthma. Although several factors have been suggested to influence this migration such as TGF-β, IL-4, IL-13, and periostin, recent evidence points to the CXCL12/CXCR4 pathway as a potential therapeutic target. Pericytes might also play an essential role in lung dysfunction in response to ageing, as they are responsive to environmental risk factors such as cigarette smoke and air pollutants, which are the main drivers of COPD. However, there is currently no direct evidence delineating the contribution of pericytes to COPD pathology. Although there is a lack of human clinical data, the recent available evidence derived from *in vitro* and animal-based models shows that pericytes play important roles in the initiation and maintenance of chronic lung diseases and are amenable to pharmacological interventions. Therefore, further studies in this field are required to elucidate if targeting pericytes can treat lung diseases.

## 1 Introduction

The lung is a crucial organ that consists of dozens of different cell populations involved in physiological processes such as gas exchange, immunity and inflammation, detoxification, and tissue repair. These cell types include epithelial cells, nerve cells, endothelial cells, immune cells, and several types of mesenchymal cells. The lung has a significant capacity for regeneration as a result of the proliferation and differentiation of progenitor cell types in response to injury (Kotton and Morrisey, 2014). One such cell type is the poorly understood and often forgotten pericyte.

Pericytes are a heterogeneous population of mesenchymal cells found within the abluminal surface of blood vessels in several tissues including the lung (Armulik et al., 2011; Shammout and Johnson, 2019). They are embedded within the basement membrane and associate closely with endothelial cells by three types of intercellular junctions: peg-socket type, gap junctions, and adhesion plaques (Caruso et al., 2009; Zhang et al., 2020).

Pericytes are emerging as an increasingly attractive cell type due to their essential roles in vascular homeostasis and remodelling, as well as in tissue injury, disease, and repair (Hung et al., 2019). Studies have shown that pericytes are essential to life, as the absence of pericytes in mice results in vascular leakage and embryonic lethality (Hellström et al., 2001). As well as preventing vascular leakage, pericytes provide structural support to capillaries and regulate contraction and vessel diameter to mediate blood pressure (pericytes expressing the contractile myofilaments α-smooth muscle actin (α-SMA) and SM22) (Bergers and Song, 2005; Hamilton et al., 2010; Barron et al., 2016; Shammout and Johnson, 2019). In addition, pericytes regulate processes such as angiogenesis, endothelial cell regulation, and immune surveillance (Hamilton et al., 2010). Therefore, it is inevitable that dysregulation of this cell type can contribute to lung diseases where pericyte-endothelial cell interactions are disrupted.

## 2 Lung pericytes

In the lung, pericytes are fundamental to maintaining the health and function of the pulmonary vasculature and thus are critical to optimal gas exchange. Depending on their location in the vascular tree (artery, capillary, or vein), pericytes control vascular tone, secrete extracellular matrix (ECM) components, regulate leukocyte extravasation, and produce mediators that maintain vascular homeostasis and angiogenesis (Shammout and Johnson, 2019). However, under conditions of acute or sustained inflammation, pericytes can also contribute to pulmonary pathology, as they are exquisitely sensitive to pro-inflammatory and pro-fibrotic mediators. This aberrant tissue microenvironment has been shown to induce pericyte uncoupling from the vessels, followed by their differentiation into myofibroblasts and remodelling of the ECM, thereby contributing to tissue fibrosis (Johnson et al., 2015; Bignold et al., 2022).

Due to their heterogeneous nature, it has proven a difficult task to characterize pericytes with definite markers owing to the difficulty in establishing robust isolation methods. This is due to differences in cell morphology as well as molecular diversity. Therefore, when isolating pericytes, it is essential to carefully choose markers to exclude similar cell types. The conventional methods used to assess the phenotype of pericytes and distinguish them from vascular smooth muscle cells (vSMC), fibroblasts, or other mesenchymal cells include flow cytometry (FACS) and immunohistochemistry (Khan et al., 2021; West et al., 2021). Reverse transcription polymerase chain reaction (RT-PCR) has also been used to compare levels of expression of gene markers, with some studies comparing pericytes markers to those of bone marrow-derived mesenchymal stem cells (MSCs) (Bagley et al., 2006; Wilson et al., 2018; Bordenave et al., 2020b; Li et al., 2021; Meng et al., 2021).

### 2.1 Lung pericyte markers

In the human lung, the most common markers used to characterize pericytes are positive markers platelet-derived growth factor receptor-β (PDGFRβ) and proteoglycan neural glial antigen-2 (NG2 or chondroitin sulphate proteoglycan 4, CSPG4), and negative markers CD31 and CD45 (Cho et al., 2003; Yuan et al., 2015; Wilson et al., 2018). Other markers used to identify pericytes from various sources and species include regulator of G-protein signalling 5 (RGS5), 3G5, CD146, CD90/thymus cell antigen 1, calponin, and intermediate filaments such as desmin and vimentin (Cho et al., 2003; Yuan et al., 2015; Hung et al., 2019).

Within the lung, other resident mesenchymal cells such as fibroblasts share a similar morphology and also express markers associated with pericytes including CD73, CD90, CD44, PDGFRα, and endoglin/CD105 (Barron et al., 2016; Hung et al., 2019). Pulmonary pericytes have also been found to highly express markers which are also expressed by MSCs such as the integrins CD29 and CD49a, and also express CD47, CD105, CD266 and Ly-51, and lysosomal degranulation markers CD107a and CD107b (Bignold et al., 2022). Fibroblasts are particularly difficult to differentiate from pericytes as there is a universal lack of specific markers isolating mural cells from fibroblasts, which complicates cell discrimination and *in situ* expression analysis (Muhl et al., 2020). Recent efforts to distinguish pericytes from other cell types have involved the single-cell RNA sequencing (scRNA-seq) analysis of fibroblasts, smooth muscle cells (SMCs), and pericytes. These studies have built foundations in the form of gene marker databases for molecular signatures of pericytes in different organs. These studies confirm a greater heterogeneity within pericytes compared to SMCs and considerable organotypicity (Muhl et al., 2020).

The markers used to characterise pericytes vary depending on the location of the cell–in terms of organotypicity, there are clear differences between brain pericytes and lung pericytes (Vanlandewijck et al., 2018). Pericytes are also specific on the vascular level and are affected by biological processes such as angiogenesis and vascular remodelling, developmental and disease states, as well as *in vitro* cell culture conditions (Armulik et al., 2011). For example, depending on their location, pericytes contain different cytoskeletal features. Pericytes that wrap around the capillary vessels of the mesentery are negative for α-SMA; however, pericytes attached to small venules are positive for α-SMA (Kapanci et al., 1992; Johnson et al., 2015). Therefore, in this respect, they may be difficult to initially distinguish from myofibroblasts. In the lung and gut, RGS5 expression is also more specific to visceral smooth muscle where it overlaps with NG2 expression, but not PDGFRβ expression (Bondjers et al., 2003). Thus, pericyte subtypes add more ambiguity to marker selection.

Using these markers, lung pericytes have been isolated primarily from humans and mice. A limited number of studies have shown that pericytes can be isolated from human lung samples using antibodies against PDGFRβ (Wilson et al., 2018), the ganglioside 3G5 (Ricard et al., 2014; Yuan et al., 2020), NG2 (Bagley et al., 2006), CD73/CD90 (Bichsel et al., 2015), or CD146 (Meng et al., 2021) *via* FACS (Bichsel et al., 2015), magnetic bead sorting (Bagley et al., 2006; Yuan et al., 2015; Wilson et al., 2018; Bordenave et al., 2020b; Li et al., 2021), magnetic beads sorting coupled with FACS (Meng et al., 2021), or cell-selective culture conditions (Yamaguchi et al., 2020). However, isolation of pericytes from lung tissue is difficult due to its fibrous nature, which results in low yields (Dore-Duffy and Esen, 2018). Thus, there is currently no gold standard for the isolation of pericytes from human lung tissue and the standardisation of protocols is required.

**FIGURE 1:**
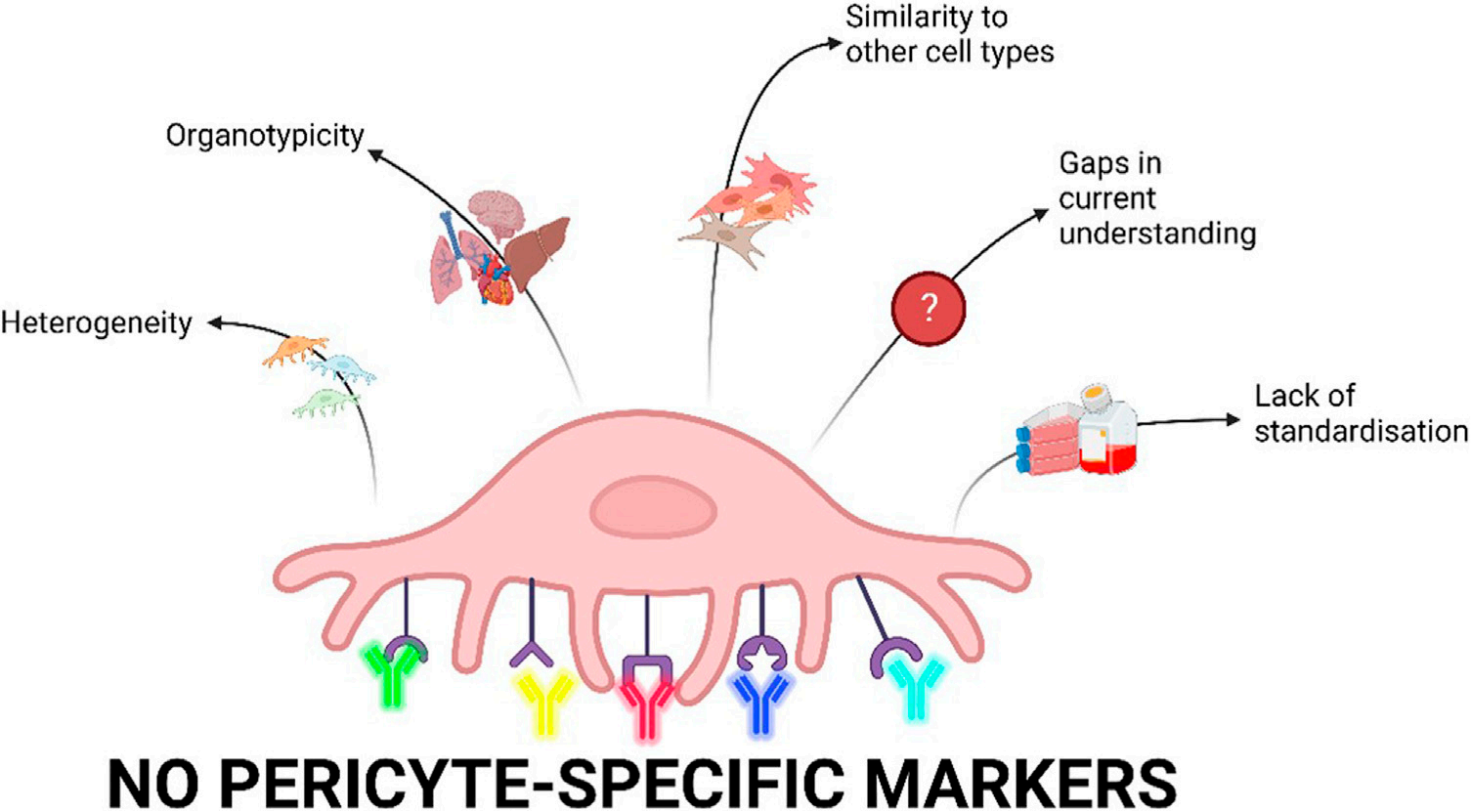
Current limitations of pericyte research. Pericytes are heterogenous depending on their location in the vasculature as well as biological processes and culture conditions. scRNA-seq data have shown major differences in pericytes derived from different organs. Furthermore, pericytes share markers with other cell types including fibroblasts, vascular smooth muscle cells, and mesenchymal cells and are therefore difficult to distinguish. Our current understanding of pericytes is limited as there are gaps in the research on pericyte physiology and pathophysiology. Finally, there is a lack of standardisation of isolation, characterisation, and cell culture conditions for pericytes which is ultimately driven by a lack of pericyte-specific markers (Created with Biorender.com).

Therefore, the heterogeneity, organotypicity, molecular diversity, and the lack of a gold standard for the isolation and characterisation of pericytes limits pericyte-related research (Figure 1). By identifying cell markers, researchers can isolate purer pericyte populations and better understand changes in different pericyte marker expression. This is useful to compare between healthy and disease states and generate biomarkers in the future (Sweeney et al., 2020; Wang et al., 2022). There is increasing awareness in the field that using scRNA seq, multipanel staining, and robust comparisons between methods will help overcome current limitations and drive research in this area.

## 3 Pericyte responses to the microenvironment

Many studies have investigated the MSC-like qualities of pericytes, such as multilineage differentiation (Crisan et al., 2008; Chen et al., 2009), phenotype (pericytes are positive for the MSC markers CD105, CD73, and CD90), and plastic adherence (Dominici et al., 2006). Recent genetic lineage tracing points to pericytes being MSC progenitor cells (Yianni and Sharpe, 2019). Thus, they have the ability to differentiate into mesenchymal cells including the hypermobile myofibroblast. It has been hypothesised that this differentiation aids the motility of the pericytes and their migration during fibrosis (Humphreys et al., 2010; Yamaguchi et al., 2020). This is supported by time course microscopy and kinetic modelling methods, suggesting that pericytes are a major source of myofibroblasts in fibrosis (Lin et al., 2008). A number of recent studies have investigated the responsiveness of pericytes to pro-fibrotic mediators, in particular TGF-β, PDGF-B, VEGF, angiopoietins, and Notch ligands. However, most of these studies are not lung-specific and thus the exact mechanisms that affect pericyte function in the lung remain unknown. Research in this area is incipient.

### 3.1 Transforming growth factor (TGF)-β signalling

Transforming growth factor (TGF)-β is an important growth factor necessary for many critical processes including lung organogenesis and homeostasis, and epithelial-mesenchymal interactions. TGF-β is also involved in regulating important responses such as tissue regeneration and immune response (Saito et al., 2018). TGF-β has long been associated with fibrotic conditions, as both the canonical and non-canonical signalling pathways have been highlighted as key mediators of fibrosis.

Induced overexpression of TGF-β in animal models has resulted in severe localised fibrosis characterised by increased ECM deposition and an increase in myofibroblast-like cells (Sime et al., 1997; Bellaye et al., 2018). TGF-β has also been thought to directly interact with pericytes during fibrosis due to the receptor for TGF-β, TGF Receptor-β (TGFR-β) being present on the cell surface (Butsabong et al., 2021). Thus, TGF-β stimulation triggers the differentiation of pericytes into scar-forming myofibroblasts in a process referred to as pericyte-to-myofibroblast transition (PMT) which is essential to wound healing. However, excess PMT is believed to be a significant contributor to fibrosis (Wu et al., 2013; Yamaguchi et al., 2020; Zhao et al., 2022). Multiple studies have explored the effect of TGF-β on the differentiation of pericytes into myofibroblasts, thusly gaining a more motile phenotype and contributing to the increased population of myofibroblasts at the site of inflammation (Johnson et al., 2015). TGF-β-induced subretinal PMT has been shown to act in Smad2/3 and Akt/mTOR pathways (Wu et al., 2013; Zhao et al., 2022). Thus, TGF-β signalling is important to pericyte function but may be dysregulated and contribute to lung fibrosis.

### 3.2 PDGF-B/PDGFRβ signalling

The most highly characterized signalling pathway that affects pericytes is the PDGF-B/PDGFRβ signalling axis, also referred to as the pericyte-endothelial signalling axis, which is essential to embryonic and organ development. PDGF-B is a growth factor secreted at high levels by angiogenic endothelial cells and at a lower level by quiescent endothelial cells and binds to PDGFRβ expressed on the surface of developing pericytes (Armulik et al., 2011). PDGFRβ knockout embryos lack pericytes and vSMCs and thus PDGFRβ expression is not specific to pericytes (Noskovičová et al., 2014). PDGFRs are activated by the binding of ligands which triggers consequent cell signalling cascades that regulate cell proliferation, differentiation, migration, mural cell coating, and survival (Thijssen et al., 2018; Del Gaudio et al., 2022).

The PDGF-B/PDGFRβ signalling axis serves as a paracrine endothelium-to-mural cells signalling loop which is initiated in the endothelium where the PDGF ligand is produced and secreted and receptor activation drives the differentiation of mesenchymal progenitor cells to mural cells and promotes mural cell coating of the blood vessels (Gaengel et al., 2009; Del Gaudio et al., 2022). PDGF-B/PDGFRβ signalling mediates the recruitment of pericytes to the endothelial cells of new vessels thus providing support and secreting additional cytokines to aid in angiogenesis (Thijssen et al., 2018). Thus, PDGFRs are linked to disease pathology, and overexpression is associated with multiple diseases including lung disease (Noskovičová et al., 2014; Johnson et al., 2015).

### 3.3 Vascular endothelial growth factor (VEGF) signalling

While the PDGF-B/PDGFRβ signalling axis and TGF-β signalling are believed to modulate processes such as pericyte recruitment, proliferation and differentiation, vascular endothelial growth factor (VEGF) is another growth factor of interest with important properties. The VEGF family is composed of five distinct ligands that bind to VEGF receptors (VEGFRs): VEGF-A, -B, -C, -D, and placental growth factor [PlGF] (Uemura et al., 2021). VEGF is crucial for embryonic vasculogenesis and angiogenesis and is considered a survival factor for cell types including endothelial cells as loss of even a single allele of the *VEGF* gene causes embryonic mortality (Ferrara, 1999). VEGFRs are primarily located on endothelial cells but also on other vascular cell types including pericytes (Takagi et al., 1996).

VEGFR1 is located on both vascular endothelial cells and pericytes, and VEGF-A acts directly on pericytes through VEGFR1 signalling. Pericyte-derived VEGFR1 has been shown to have a crucial role in cerebrovascular formation and maintenance by stabilising brain vascular integrity and promoting pericyte coverage. Through paracrine VEGFR1 signalling, pericytes regulate brain endothelial tube formation, and intracellular VEGFR1-mediated signalling is required for pericyte migration and regulated Akt signalling in pericytes (Gong et al., 2022). An *in vivo* model of cancer-associated retinopathy as well as an *in vitro* co-culture model of the blood brain barrier found that VEGFR1 induces pericyte ablation causing increased retinal vascular permeability (Cao et al., 2010; Salmeri et al., 2013). VEGF-A is a key modifier of endothelial cell sprouting and proliferation and is mediated in a pericyte-dependent fashion through VEGFR1 (Eilken et al., 2017). Studies have shown that VEGF regulates contraction and relaxation in isolated microvascular rat pericytes (Donoghue et al., 2006). Thus, VEGF-A is an important mediator of pericyte and endothelial cell function, however, the main role of VEGF-A and VEGFR1 on pericytes in the lung awaits further investigation.

### 3.4 Angiopoietin signalling

In contrast to the PDGF-B/PDGFRβ signalling axis, the Angiopoietin/Tie (Ang/Tie) signalling mainly represents a signalling loop from mural cells to the endothelium and acts as a key regulator of adult vascular homeostasis (Augustin et al., 2009; Gaengel et al., 2009). Tie receptors are receptor tyrosine kinases activated by Ang ligands. Functional Tie2 receptors are expressed in low levels on various human and murine pericyte subtypes including lung pericytes. Tie receptors are stimulated in a paracrine fashion by Ang1 which is secreted by pericytes and other cells and are modulated by Ang2 which is mainly secreted by endothelial cells (Teichert et al., 2017). Therefore, in combination with the PDGF-B/PDGFRβ signalling between pericytes and endothelial cells, Tie2 on endothelial cells can bind to Ang1 produced by pericytes which results in a reduction of gaps between endothelial cells and therefore reduced vascular leakage (Baffert et al., 2006; Fuxe et al., 2011). Via this mechanism, pericytes can influence the influx of materials in and out of the bloodstream and contribute to vascular maturation.

Tie2 signalling has also been linked to angiogenesis and survival signals through Calpain, phosphoinositide 3-kinase (PI3K)–Akt and FOXO3A (Augustin et al., 2009; Teichert et al., 2017). In sprouting angiogenesis, pericyte-produced Ang1 stabilizes stalk cells *via* Tie receptor complexes which promotes cell survival, matrix interactions, and endothelial barrier function (Jeltsch et al., 2013). Conversely, endothelial cell-secreted Ang2 has paracrine effects on pericytes, and may compete with Ang1 to contribute to vascular destabilization (Teichert et al., 2017). *Ng2-Cre*-driven deletion of pericyte-expressed Tie2 delays developmental angiogenesis and vessel maturation in mice, and Tie2 deletion in pericytes results in a pro-angiogenic tumour vasculature with enhanced tumour growth. Furthermore, silencing Tie2 expression significantly increases brain pericyte motility (Teichert et al., 2017). Ang signalling may also affect pericytes in a Tie2-independent fashion. For example, in a study on diabetic mouse retinas, Ang2 increase induced p53-dependent pericyte apoptosis *via* integrin signalling (Park et al., 2014). Therefore, lineage tracing using Tie2 as a driver also labels pericytes which can be used to inform experimental design in future.

### 3.5 Notch signalling

Notch signalling is essential for early stage pericyte development in zebrafish (Ando et al., 2019). Studies have shown that Notch signalling regulates mural cell differentiation and function and that pericytes predominately express Notch3, but also express Notch1, and low levels of Notch2 and Notch4 (Vanlandewijck et al., 2018; Nadeem et al., 2020; Bignold et al., 2022). In studies using retinal pericytes, it was proposed that Notch signalling is crucial to pericyte survival as they highly express canonical Notch/RBPJK (recombination signal-binding protein one for J-kappa) downstream targets and activation of Notch signalling reduced light-induced cell death (Arboleda-Velasquez et al., 2014). The mechanism behind Notch-mediated pericyte survival was determined to be regulation of PDGFRβ levels (Nadeem et al., 2020). However, there is conflicting evidence surrounding how Notch3 function affects pericytes depending on the model investigated–Notch3^−/−^ mice (Henshall et al., 2015), diabetic Notch3^−/−^ mice (Liu et al., 2018), zebrafish (Wang et al., 2014), or in patients with cerebral autosomal dominant arteriopathy with subcortical infarcts and leukoencephalopathy (CADASIL) (Dziewulska and Lewandowska, 2012; Del Gaudio et al., 2022). Therefore, further investigations into how Notch signalling affects pericytes is needed.

## 4 Pericyte-matrix interactions

As described above, pericytes interact with endothelial cells and can differentiate into scar-forming myofibroblasts, leading to collagen deposition and matrix remodelling and thus driving tissue fibrosis. Depending on the context and disease, these fibrotic processes are driven at different sites. For example, in asthma, fibrosis arises in the subepithelium, while in pulmonary hypertension (PH), the fibrosis occurs in the pulmonary arteries (Hamid, 2003; Simonneau et al., 2019). Pericyte-ECM interactions as well as other biochemical cues such as growth factors and cytokines play crucial roles in these processes. The way in which cells interact with their surrounding microenvironment is complex, however, it is evident that changes in the ECM affect cellular responses (Cox and Erler, 2011). The mechanical features and mechanobiology of pericytes have been gaining important traction in recent years (reviewed recently by (Dessalles et al., 2021)). Due to the role of pericytes in the formation and maintenance of the vasculature, various forces including contractile and mechanical forces are critical aspects that affect pericyte function. The various subtypes of pericytes have different morphology, localization, as well as mechanics and thus these factors, alongside the physiology of the local microenvironment, should be considered when creating 2D and 3D *in vitro* models incorporating pericytes (Dessalles et al., 2021). Further insights into pericyte-ECM interactions are needed to advance our understanding of the biophysical and biochemical cues which affect pericyte behaviour and function and, conversely, how pericytes affect the ECM, for example, in terms of stiffness.

### 4.1 Disruption to pericyte signalling in disease

Pericyte migration occurs in healthy tissue as a way for recruited pericytes to move towards newly formed blood vessels and endothelial sprouts in order to maintain coverage. During this process, pericytes can be observed migrating linearly along the existing blood vessels whilst maintaining contact with the endothelial cell layer suggesting that this migration is mainly controlled by endothelial cell interactions (Payne et al., 2021). This is in stark contrast to the migration observed in inflamed tissue, as this is associated with the uncoupling of pericytes from endothelial cells as a primary event and therefore is likely to be influenced by separate factors.

Disruption of pericyte-endothelial interactions by drugs or by cytokine changes occurring in inflammation can cause the dissociation of pericytes from the vasculature. This can lead to weakened blood vessel structures, resulting in hyperdilation and low blood pressure (Abramsson et al., 2003). It can also cause vascular leakage, which can exacerbate inflammation as well as leave the tissue vulnerable to bloodborne pathogens such as the Dengue virus (Cheung et al., 2020). Once detached from the blood vessels, pericytes may continue to affect the surrounding tissue in specific ways. Within inflamed lung tissue, it has been observed that pericytes migrate chemotactically into inflamed areas, i.e., the large conducting airways in asthma and inflamed pulmonary arteries in pulmonary arterial hypertension (PAH) (Johnson et al., 2015; Bordenave et al., 2020a). Understanding the factors causing the directed migration of pericytes under these conditions is an area of active research. Moreover, modifying the specific factors that contribute to the pericyte migration observed in inflammatory/fibrotic conditions is a viable pharmacological strategy to alleviate the migration of pericytes and therefore reduce their contribution to organ fibrosis. Despite this, it is not yet clear which factors have the greatest impact on pericyte migration and therefore it is yet to be elucidated which would be the best targets for therapeutic action.

## 5 Pulmonary hypertension (PH)—Symptoms, disease course, and pathological mechanisms

PH manifests with symptoms including dyspnoea, fatigue, dizziness, angina, irregular heartbeat, and oedema and is clinically defined as abnormally high pressure in the pulmonary artery and right ventricle of the heart, i.e., above 25 mmHg at rest according to current guidelines (Simonneau et al., 2019). A number of pathologies can lead to PH, with five types of PH defined by the World Health Organization: pulmonary arterial disease, left heart disease, lung disease, chronic thromboembolic disease, and unclear or multifactorial mechanisms (Wu X. H. et al., 2022). Of these, Type 1 (PAH) results from endothelial cell dysfunction, leading to inflammation, occlusive remodelling of the small pulmonary arteries, accumulation of vascular smooth muscle cells, and fibrosis; these processes ultimately lead to the formation of plexiform lesions in the pulmonary arteries and right ventricular failure (Liu et al., 2022). The 3-year mortality rate for PAH patients is currently 21% (Chang et al., 2022).

Inflammation of the pulmonary vasculature is currently understood to be a common characteristic of several types of PH, predominantly observed in the pulmonary adventitia (Savai et al., 2012). In the lungs of PAH patients, this inflammatory response is characterized by the infiltration of macrophages, mast cells, dendritic cells, and CD8^+^ cytotoxic T cells (Savai et al., 2012). Immune cell infiltration into the vessel wall subsequently initiates a dysregulated wound healing process, leading to excess ECM production and vessel wall calcification. The loss of pulmonary artery compliance as a consequence of these structural changes to the vessel wall increases right ventricular afterload and has been shown to be a sensitive predictor of mortality in PAH (Papolos et al., 2021).

The role of pericytes/vSMCs in driving PAH pathology is an area of active investigation. A recent study by Crnkovic et al. (2022) using scRNA-seq of healthy and PAH human lungs demonstrated the very high transcriptional similarity between pericytes and SMCs (Crnkovic et al., 2022). Overall, this study demonstrated a broad range of functional and phenotypic changes in pericytes and vSMCs in PAH. In particular, these authors showed that PAH lung pericytes represent a contractile, α-SMA expressing cell type, indicating the capacity of pericytes to differentiate into myofibroblasts and thereby contribute to vascular wall thickening and reduced vascular lumen diameter in the PAH lung. Further analysis of the data revealed an increased capacity for oxygen sensing in pericytes obtained from remodelled PAH vessels, with a notable increase in *NDUFA4L2* which is a mitochondrial electron chain protein that has been associated with hypoxia-induced PH (Liu et al., 2021).

Understanding the cellular source of ECM proteins (collagens I, IV, and XIV, fibronectin, tenascin-C) in PAH has long been an area of intensive research (Botney et al., 1993; Ihida-Stansbury et al., 2006; Wei et al., 2012; Hoffmann et al., 2015). Moreover, extensive analysis of clinical samples has demonstrated increased proliferation and accumulation of vSMCs in remodelled pulmonary arteries (Sakao et al., 2010; Mura et al., 2019). Although sophisticated *in vitro* vessel-on-chip constructs have been designed and implemented to interrogate the mechanisms of these processes (Ainscough et al., 2022), a full mechanistic evaluation of crosstalk between multiple structural and immune cell types and the factors driving vSMC proliferation and ECM deposition still requires the use of animal models of PAH in order to identify promising new therapeutic targets.

### 5.1 Preclinical models of pulmonary arterial hypertension (PAH)

A number of animal models of PH have been developed in an effort to recapitulate the mechanistic origins and pathophysiology of clinical PH. These animal models (predominantly established in rats and mice) can be defined according to the method of model induction: non-invasive, invasive, and genetically modified models (Wu H. et al., 2022). However, as the molecular mechanisms of clinical PH are not fully understood, particularly in the case of idiopathic PAH, these models cannot be relied upon to explain early events in PH induction and progression. Despite this, the pathophysiological outcomes of many of these models closely recapitulate human disease, suggesting that the knowledge gained from these models may be useful in the pursuit of novel therapeutic targets.

Non-invasive models of PH in rodents are primarily driven by chronic hypoxia (CH), treatment with the VEGFR-2 antagonist Sugen 5,416 (SU5416; semaxanib), or exposure to the macrocyclic pyrrolizidine alkaloid monocrotaline (Wu H. et al., 2022). In CH-induced PH rats, exposure to 10% oxygen for a period of 3–4 weeks results in pulmonary arterial remodelling and endothelial cell dysfunction (Wu et al., 2020); similar CH exposure in mice results in a milder phenotype (West and Hemnes, 2011). These changes are reversible once the animal is returned to a normoxic environment, so these models best recapitulate milder PH phenotypes such as those associated with lung diseases such as chronic obstructive pulmonary disease (COPD) and interstitial lung disease (ILD) (Nathan et al., 2019). In SU5416-driven models of PAH, with concomitant CH, both mice and rats demonstrate vigorous pulmonary endothelial cell proliferation, vascular remodelling with plexiform lesions, and PH, even after the animal has been returned to normoxia (Abe et al., 2010). Important considerations should be taken into account when using these models regarding animal strain (Lewis rats are only minimally responsive whereas Sprague-Dawley rats manifest with severe disease) (Jiang et al., 2016) and gender (female sex hormones may protect against the initiation of disease) (Chaudhary et al., 2021). Despite the fact that the inflammatory component of PH pathology may not be fully recapitulated, SU5416/CH models have been used for over 20 years in preclinical investigations into PAH therapeutics. Conversely, the monocrotaline (MCT) model of PH has a strong inflammatory component, having first been shown in the 1960s to induce vascular endothelial cell damage and pulmonary arteritis in rats, ultimately leading to pulmonary vascular remodelling and right ventricle hypertrophy (Kay et al., 1967). Since then, this model has been refined and adapted to study neonatal and obesity-associated PH, as well as the impact of the gut microbiome on PH (Kazama et al., 2014; Hong et al., 2021; Lesage et al., 2021). Importantly, the degree of cytotoxicity induced by MCT in organs other than the lung cannot be ignored, as this does not reflect the situation in clinical PH.

Invasive surgical models of PAH are rarely used due to their technical complexity and high mortality rate; however, these models can be useful to dissect the mechanisms of right ventricular overload and failure. These invasive methods can include pneumonectomy, the insertion of a pulmonary shunt, or pulmonary artery binding, which increase blood flow and pressure in the pulmonary arteries, a key characteristic of PH. Combining surgical methods (usually removal of the left lung) with non-invasive means of inducing PH (e.g., SU5416 or MCT) induces a phenotype closer to that seen in human PAH, with both increased blood pressure in the remaining right lung along with endothelial damage induced by post-surgery MCT or SU5416 treatment (Katz et al., 2019). Despite the high degree of accuracy in reproducing human disease characteristics, the high degree of surgical skill required has limited the widespread adoption of these combined models.

The advent of gene editing technology has led to the broad proliferation of PH models based on genetically manipulating the inflammatory and biochemical mechanisms thought to initiate the development of PH. A number of genes found to predispose humans to the development of PH, i.e., *BMPR2*, *KCNK3*, and *SMAD9*, as well as inflammatory genes associated with endothelial dysfunction (IL-6 and hypoxia-inducible factor) have been targeted to establish these models, with varying levels of success in inducing vascular inflammation, remodelling, and dysfunction (reviewed in detail by Dignam et al., (2022)). However, as most of these models are induced in mice, a species that does not readily develop the full range of pathology observed in humans, a second stimulus (e.g., hypoxia (Hilton et al., 2022) or cigarette smoke exposure (Heydarian et al., 2022)) is usually needed to establish a severe disease phenotype. Crucially, without a deeper understanding of the initiating factors driving PAH in humans, these mouse models will remain only an approximation of the clinical form of the disease; this may have important implications in the delineation of potential therapeutic targets.

### 5.2 Recent developments on the role of pericytes in PAH pathology

Several recent studies using preclinical models of PAH have demonstrated that lung pericytes are major contributors to PAH pathology, with major insights provided by women working in this field of research.

In an elegant study employing primary human lung pericytes and a SU5416/CH-driven model of PAH in transgenic mice bearing GFP-labelled pericytes, Bordenaeve et al. (2020b) demonstrated the acquisition of functional and phenotypic aberrations in pericytes under disease conditions. These investigators further demonstrated that the increase in migratory capacity observed in PAH pericytes was mediated by the CXCR7/CXCR4-CXCL12 pathway. Conversely, the acquisition of a myofibroblast phenotype by pericytes was dependent on increased TGF-β signalling *via* the upregulation of TGFβRII. Promisingly, further investigations showed that neutralizing the activity of the chemokine CXCL12 (also known as SDF1) using the naturally occurring neutraligand chalcone 4 was able to suppress early pericyte accumulation at the pulmonary arterioles of mice, suggesting that the CXCL12 pathway may be a viable target for the treatment of PAH. Similarly, using lineage tracing for pericytes in a CH-driven PAH model, Yuan et al. (2020) showed that pericytes under diseased conditions become more responsive to CXCL12 and contribute to the muscularization of small pulmonary arterioles. Intriguingly, the genetic ablation of CXCL12 specifically in NG2−positive pericytes resulted in significantly reduced vascular pathology following exposure to hypoxia compared with wild-type mice, which showed increased right ventricular systolic pressure, right ventricular hypertrophy, and vascular muscularization. Similar results were seen following the pharmacological blockade of CXCL12 activity using the CXCR4 antagonist AMD3100, again highlighting the importance of CXCR4 activity in controlling the migratory capabilities of pericytes.

In another study, Yuan et al. (2020) were also able to show that, beyond the muscularization of pulmonary arterioles, pericyte dysfunction may also contribute to the loss of microvessels in the lung following exposure to CH. In these experiments, the authors demonstrated that the PAH-associated loss of Wnt5a in endothelial cells prevented pericyte recruitment and angiogenesis, which not only impaired vascular regeneration after a period of hypoxia but may have also freed up pericytes to subsequently transform into myofibroblasts and contribute to large vessel muscularization. It will be interesting to determine if altered Wnt5a expression and aberrations in the planar cell polarity pathway play important roles in the repair of the pulmonary vasculature after injury and in other diseases where altered endothelial cell/pericyte interactions are an underlying pathology.

In a similar vein, the research group of Karin Tran-Lundmark have demonstrated that altered PDGF-B signalling in the lung can protect against the development of vascular muscularization in response to hypoxia (Tannenberg et al., 2018). In these experiments, considering that homozygous knockout of PDGF-B or its cognate receptor PDGFRβ is embryonically lethal, Pdgfb^ret/ret^ mice were used to downregulate PDGF-B activity–loss of the retention motif on the C-terminus of PDGF-B allows this growth factor to freely diffuse throughout the tissue rather than being retained in the vascular basement membrane where it exerts its activity of recruiting pericytes to endothelial cells. In the context of reduced PDGF-B activity, hypoxia-induced pulmonary vessel muscularization was highly disorganized and less severe than in wild type hypoxic mice, resulting in the normalization of hemodynamic parameters and reduced disease severity. Although the pharmacological blockade of PDGFRβ is possible using compounds such as imatinib, the promiscuous nature of receptor tyrosine kinases and their widespread expression are associated with detrimental side effects, suggesting that more targeted strategies are necessary to pursue PDGF-B as a therapeutic target in PAH (Hoeper et al., 2013).

Additional insight into the critical importance of pericyte-endothelial cell interactions in PAH was provided in a study by Wang et al., showing that the loss of prolyl hydroxylase domain protein 2 (PHD2) in endothelial cells increased pericyte recruitment to vessels and led to PAH in the absence of any additional insult (Wang et al., 2016). Endothelial cell-specific knockdown of PHD2, which has the function of degrading hypoxia inducible factor-α (HIF-α), increased the expression of TGF-β in pulmonary pericytes and promoted perivascular fibrosis, right ventricular hypertrophy, and pulmonary vessel dysfunction. Mechanistically, these changes were mediated by aberrant Ang1/Notch3 signalling, as PHD2-deficient endothelial cells produced higher levels of Ang1, thereby increasing the expression of Notch3 and promoting pericyte proliferation and excessive pericyte coverage of pulmonary vessels.

Considering the fact that Yuan et al. (2020) showed that pericytes obtained from the lungs of PAH patients expressed significantly higher levels of CXCL12 compared to healthy lung pericytes, attenuation of the CXCL12/CXCR4 pathway holds considerable promise. Indeed, Bordenave et al. (2020a) have shown that the daily treatment of rats with established PAH (induced either by MCT injection or SU5416-CH) with compounds that suppress CXCR4 activity was able to reduce PAH disease severity, attenuate right ventricular hypertrophy, prevent remodelling of the pulmonary vasculature, and decrease pericyte coverage of arterioles. Interestingly, the use of neutraligands against the chemokine CXCL12 (chalcone 4 or the more bioavailable LIT-927) was more effective than the pharmacological blockade of the receptor CXCR4 with AMD3100. It is anticipated that further refinements to CXCL12 neutraligands may be beneficial in the treatment of PAH, not only in terms of preventing vascular remodelling but also by attenuating the infiltration of CXCR4+ macrophages, which may provide an additional clinical benefit.

Beyond these studies performed in mouse models of PAH, recent work has provided novel insight into the genetic basis of pericyte dysfunction in PAH (Pienkos et al., 2021). By performing whole genome sequencing on PAH patients with a family history of the disease, these authors revealed deficits in *TNIP2* and *TRAF2*, which encode proteins that regulate the activity of NF-κB, previously associated with inflammation and vascular remodelling in PAH. Indeed, knockdown of these two transcripts in human pericytes led to a substantial increase in pericyte proliferation. It is anticipated that additional genomic studies of PAH patients will reveal other pathways that alter pericyte homeostasis in this disease.

## 6 Allergic asthma—Symptoms, disease course, and pathological mechanisms

Asthma is an incredibly common lung disease currently affecting around one in 12 people in the UK (British Lung Foundation, 2023) and has been the subject of an abundance of research in previous years. Despite this, the extent to which pericytes contribute to the characteristic airway remodelling seen in allergic asthma, as well as the disease as a whole is still debated. The pathology of allergic asthma is initiated by the inhalation of an allergen which commences a cascade of cytokines and physiological changes resulting in the remodelling of the airway. This remodelling includes; epithelial loss, mucus gland hyperplasia, subepithelial fibrosis, inflammatory cell infiltration, bronchial smooth muscle hypertrophy and vascular changes (Hamid, 2003). These all result in a hyperresponsive airway and airway obstruction causing the coughs and wheezes synonymous with asthma. Pericytes have been long thought to contribute to the pathophysiology of asthma, although the extent to which they contribute is highly contested. However, pericytes have been shown to affect every element of airway remodelling both directly and indirectly making their importance in allergic asthma undeniable. The plasticity of pericytes combined with their penchant for both producing and responding to cytokines increases their scope of effect and makes them a highly favourable element in future drug discovery.

### 6.1 Preclinical models of asthma

Unlike humans, mice do not naturally develop allergic asthma, but under controlled conditions can develop Th2- or Th17-polarized airway inflammation in response to respiratory allergen exposure (recently reviewed in detail (Rydell-Törmänen and Johnson, 2019)). The principal considerations in the design of animal models of human disease are the aetiology and the presentation of the model, i.e., the method of disease induction and how the disease develops over time (Mullane and Williams, 2014; Williams and Roman, 2016). Ideally, the features of human disease should be found in a physiologically relevant mouse model; in the case of allergic asthma, these include respiratory allergen exposure, a complex immune response coordinated in the bronchopulmonary lymph nodes, inflammation and remodelling of the large airways, and airway hyperresponsiveness (wheezing) (Holgate et al., 2015). Since early mouse models of asthma using the surrogate protein ovalbumin (OVA) failed to recapitulate these features (Alessandrini et al., 2020), this field of research has moved on to using mouse models of allergic asthma driven by respiratory environmental allergen exposure. These novel models more closely replicate the route of allergen exposure, the site of disease induction, and the structural and physiological consequences of chronic allergic asthma as seen in the clinic.

The most commonly used allergen-driven model employs house dust mite (HDM), which has inherent allergenic properties and induces severe asthma-like airway inflammation, prominent airway wall remodelling, and airway hyperresponsiveness (Johnson et al., 2004; Bignold et al., 2022). Other models employ additional clinically important environmental allergens, such as cockroaches, *Alternaria*, and pollens, either singly or in combination (Arizmendi et al., 2011; Wimmer et al., 2015; Yee et al., 2018; Lewis et al., 2022). These models were developed to be clinically relevant, and the immunological responses to these allergens are relatively similar to what is seen in asthmatics (Rydell-Törmänen and Johnson, 2019). However, there are important physiological and pharmacological differences between mice and humans that need to be taken into consideration when attempting to translate the results of mouse studies into clinical trials.

### 6.2 Pericytes and the regulation of immune cell infiltration

A key step to the progression of asthma as a disease is increased immune cell infiltration. Asthma is often thought of as a Th2 inflammatory condition with the majority of asthmatics having an increased Th2 response to airway allergens (León and Ballesteros-Tato, 2021). Many current treatments for asthma attempt to modulate this response, including inhaled corticosteroids, omalizumab, and several inhibitory antibodies targeting Th2 cytokines such as IL-4, IL-5 and IL-13 (Bleecker et al., 2016; Hossny et al., 2016; Kawakami and Blank, 2016; Castro et al., 2018). Many cases of asthma are also eosinophilic, often with the extent of the eosinophilia determining disease severity (Bakakos et al., 2019). The Th2 cytokines IL-5, IL-4 and IL-13 have all been linked to eosinophil trafficking or the activation of adjacent signalling pathways such as the IgE cascade responsible for Th2 lymphocyte production thus encouraging further migration of eosinophils (Fulkerson and Rothenberg, 2013; Pelaia et al., 2022). In addition to increases in eosinophils, many other immune cells are also upregulated within inflamed airways including dendritic cells, basophils and mast cells which may also have antigen-presenting roles alongside their effector roles (Kim et al., 2010).

**FIGURE 2:**
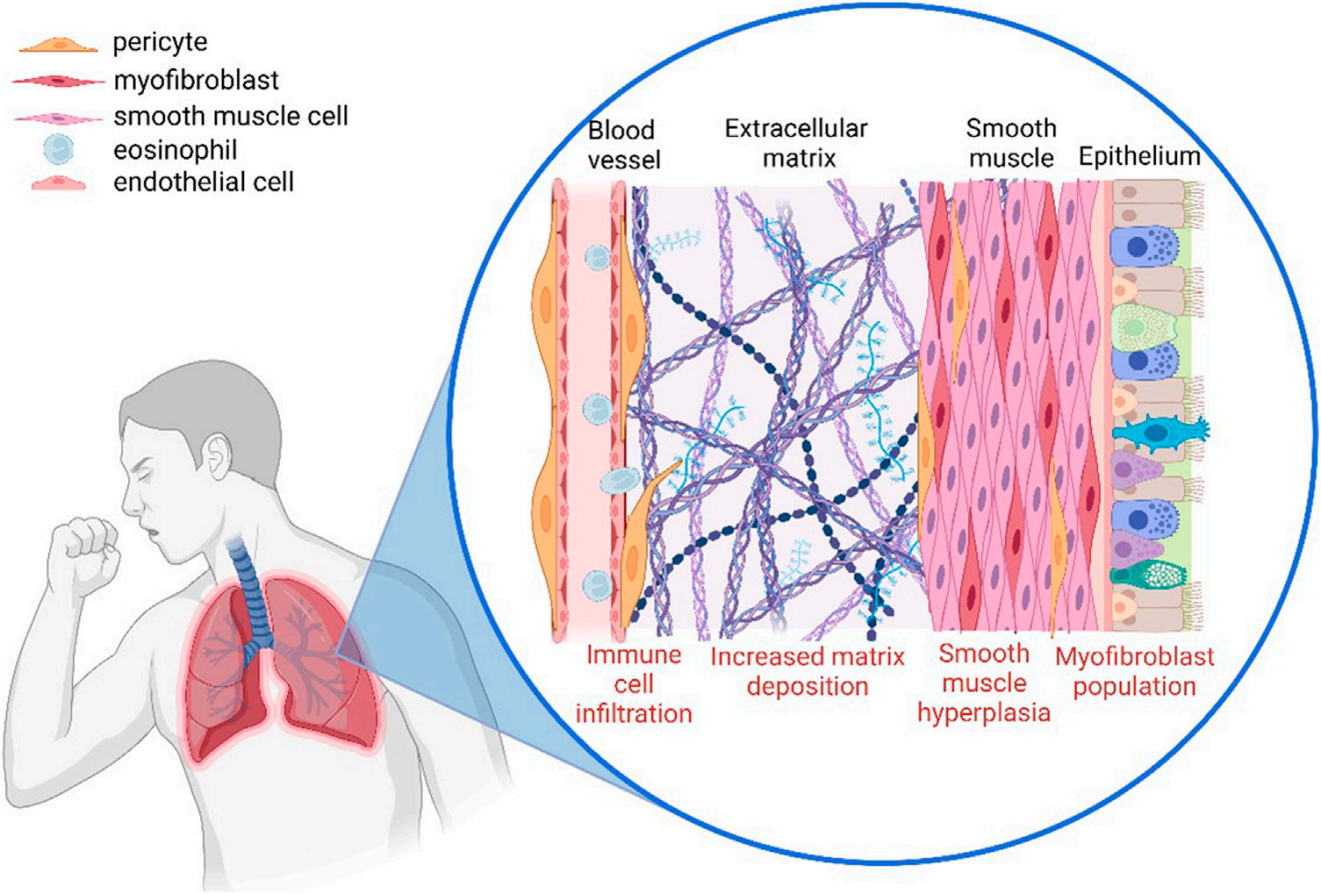
The involvement of pericytes in the airway remodelling observed in asthmatic lungs. Pericytes modulate immune infiltration, detach from the blood vessel and migrate towards the site of inflammation where they increase deposition of extracellular matrix components, and also contribute to vascular leakage (Created with BioRender.com).

Pericytes play a critical role in controlling the infiltration of immune cells from the bloodstream towards the site of inflammation. It has been observed that when pericytes migrate away from blood vessels towards the site of inflammation, they leave gaps which can result in vascular leakage and unregulated immune cell infiltration (Figure 2) (Ferland-McCollough et al., 2017). There is also increasing evidence that pericytes play an active role in the recruitment of immune cells *via* the secretion of cytokines such as CXCL1 and CXCL8 as well as the induction of vasodilation which would increase the rate of infiltration (Navarro et al., 2016). This highlights the importance of pericytes in both the initiation and the progression of inflammation.

### 6.3 Contribution of pericytes to subepithelial fibrosis in allergic asthma

Subepithelial fibrosis is probably the most apparent structural change that occurs during asthma. Many studies have shown that asthmatic airways contain remodelled basement membranes which alter the properties of the airways. One of the changes in the subepithelium is the increase in ECM proteins. This is likely caused by aberrant myofibroblasts and can include a range of proteins including several types of collagen and adhesion proteins such as fibronectin (Ito et al., 2019). The increase in these proteins would encourage cross-linking, leading to a stiffer, overly responsive membrane (Jamieson et al., 2021). Within the asthmatic subepithelium, there is also an alteration to the SMC population. Hyperplasia and hypertrophy of airway SMCs is often found within asthmatic airways. Hypertrophy of SMCs increases their contractility and therefore contributes to airway hyperresponsiveness. It has also been suggested that hypertrophic smooth muscle may have a protective effect on neighbouring myofibroblasts by modulating apoptosis (Chetty and Nielsen, 2021). The increased number of SMCs also increases the contraction of airways as well as the responsiveness of the cells to stimuli (Khan, 2013). SMCs can also contribute to the increased ECM deposition previously explained, thus increasing their contribution to airway stiffness (Cheng et al., 2016). Similar to SMC hyperplasia, there is also an increase in the myofibroblast population around asthmatic airways (Boser et al., 2017). Myofibroblasts contribute to a large amount of ECM deposition as well as contractile forces, thus exacerbating airway stiffness and hyperresponsiveness (Darby et al., 2014).

Our research group has demonstrated that pericytes largely contribute to subepithelial fibrosis in allergic asthma due to their ability to differentiate into myofibroblasts. Due to their contractile ability, when pericytes migrate towards the airway wall during inflammation, they contribute to the increased contractile forces produced by SMCs (Johnson et al., 2015). In addition, since they express similar markers (α-SMA, SM22), pericytes may actually be included in the increased cell counts observed in smooth muscle hyperplasia (Smyth et al., 2018). It has also been suggested that the differentiation of pericytes into myofibroblasts contributes to much of the increased myofibroblast population in inflamed airways (Johnson et al., 2015). Therefore, pericytes can contribute both to increased airway constriction and aberrant ECM deposition within fibrotic airways.

### 6.4 Therapeutic strategies targeting pericytes in allergic asthma—*CXCL12*

CXCL12 is a ligand which binds to the receptor CXCR4 that is found on pericytes. It has long been associated with the bone marrow and the retention of hematopoietic stem cells within the stem cell niche (Kim and Broxmeyer, 1998). As similarities between pericytes and these progenitor cells have been observed, as well as the presence of the receptor on the pericyte surface, CXCL12 has also been linked to the migration of pericytes (Xu et al., 2020). The overproduction of CXCL12 is currently associated with a variety of fibrotic lung diseases including idiopathic pulmonary fibrosis, COPD and asthma (Negrete-García et al., 2010; Jaffar et al., 2020; Kothapalli et al., 2021). Negrete-Garcia et al. suggested that CXCL12 was significantly elevated within the bronchoalveolar lavage samples from asthmatic patients (Negrete-García et al., 2010). As CXCL12/CXCR4 interactions also mediate pericyte-endothelial association, the increased concentration of CXCL12 at the site of inflammation may act as an attractant, facilitating the migration of pericytes towards the inflamed airway (Takabatake et al., 2009).

The inhibition of the CXCL12/CXCR4 gradient has been explored several times in the context of combating fibrosis as well as in the field of oncology (Guo et al., 2016). One of the most common inhibitors explored is AMD3100, a potent CXCR4 antagonist first developed to combat HIV (De Clercq, 2015). Studies have shown that using AMD3100 to disrupt the CXCL12/CXCR4 gradient can lead to a reduction of migration of mesenchymal cells and therefore a reduction of cells contributing to fibrosis within the lung (Lukacs et al., 2002; De Clercq, 2015; Li et al., 2020). However, it has also been seen that inhibiting CXCR4 may have unwanted effects within the bone marrow. Yang et al. observed that treatment of renal fibrosis with AMD3100 caused an infiltration of T cells from the bone marrow towards the site of fibrosis and caused increased tissue damage (Yang et al., 2016). Kumar and Ponnazhagan (2012) have similarly suggested that AMD3100 can cause the mobilisation of MSCs which, in the case of lung fibrosis, would contribute to the pool of dysregulated pericytes exacerbating the fibrosis. For this reason, we have previously explored the use of a CXCL12 inhibitor to disrupt the gradient by alternate means. LIT-927 is a neutraligand which binds to CXCL12 and prevents it from interacting with CXCR4 (Regenass et al., 2018). We have shown that treatment with LIT-927 reduces airway remodelling in a mouse model of asthma by reducing the migration of pericytes (Bignold et al., 2022). This highlights both CXCL12 and CXCR4 as druggable targets for future experimentation.

### 6.5 Therapeutic strategies targeting pericytes in allergic asthma—Periostin

Periostin is a small matricellular protein which has several roles within the ECM. It has long been linked to allergic asthma as serum periostin is often used as a biomarker for disease severity (Izuhara et al., 2016). Elevated expression of periostin has also been linked to a variety of lung diseases including idiopathic pulmonary fibrosis, interstitial pneumonia and non-small cell lung cancer (Okamoto et al., 2011; Naik et al., 2012; Okazaki et al., 2018). It has been seen to encourage migration through the upregulation of TGF-β and differentiation to a myofibroblast phenotype (Uchida et al., 2012). It can activate several key signalling pathways such as NF-κB, PI3K/Akt and FAK highlighting the widespread impact of periostin (Conway et al., 2014). This may pose the problem of off-target effects when directly inhibiting periostin with a therapeutic.

Despite this, several studies have been completed showing that periostin-null mice have significantly less fibrosis than the wild type and therefore indicate the merit in inhibiting periostin therapeutically (Uchida et al., 2012; Hwang et al., 2017). OC-20 is an antibody which has been used to directly inhibit periostin *via* the FAS1-2 domain of the protein (Orecchia et al., 2011). It has been shown to reduce airway hyperresponsiveness, cell proliferation and collagen deposition within fibrotic lung diseases (Naik et al., 2012; Bentley et al., 2014; Yamato et al., 2021). Lebrikizumab is another antibody inhibitor of periostin, although instead of directly binding to periostin, it instead binds to IL-13 and prevents the formation of the IL-13Rα/IL-4Rα dimer which induces periostin production (Guttman-Yassky et al., 2020). In clinical trials, lebrikizumab reduced the rate of asthma exacerbations as well as increased lung function in moderate-to-severe asthmatics (Hanania et al., 2015). In *in vivo* studies, it has also decreased airway hyperresponsiveness as well as reduced the amount of immune cells and pro-inflammatory mediators present in the lung (Hacha et al., 2012). Another indirect way of inhibiting periostin is using cinnamaldehyde, a compound that gives cinnamon its flavour and odour, which is shown to interact with periostin through the modulation of the Nrf2 pathway (Mitamura et al., 2018). Some studies have suggested that, *in vivo*, cinnamaldehyde can reduce both IL-13-dependent and TGF-β-dependent periostin expression (Mitamura et al., 2018). It has also been shown that cinnamaldehyde mitigates increased pericyte migration caused by periostin as well as reducing IL-13-induced production of periostin by pericytes (Bignold and Johnson, 2021). This shows that important investigation into the use of periostin as a target for pharmacological intervention is underway and may yield useful drugs to treat fibrotic lung diseases in the future.

## 7 Chronic obstructive pulmonary disease (COPD)—Symptoms, disease course, and pathological mechanisms

COPD is a complex and heterogenous syndrome characterized by progressive and incompletely reversible airway obstruction, in some patients associated with emphysema, caused by abnormalities of the airways (bronchitis, bronchiolitis) and/or alveoli (emphysema) (Olajuyin et al., 2019; Vasilescu et al., 2019). The growing number of COPD cases in recent years is due to the pandemic of tobacco smoking, environmental pollution, and ageing of the global population (Jamieson et al., 2020; Rao et al., 2020; Hiemstra et al., 2021). Though the pathobiological mechanisms of COPD remain incompletely understood, various contributors such as the protease-antiprotease imbalance, the oxidant-antioxidant imbalance, cellular senescence, autoimmunity, chronic inflammation, and defective lung growth and development are suggested to play essential roles in COPD pathogenesis (Katayama et al., 1988; Rao et al., 2020).

COPD patients typically complain of dyspnoea, activity limitation and/or cough with or without sputum production and may experience acute respiratory events characterized by increased respiratory symptoms called exacerbations that require specific preventive and therapeutic measures (GOLD, 2023). The therapeutic intervention with the greatest impact on COPD is smoking cessation. Inhaled corticosteroids (ICS) and long-acting bronchodilators, including long-acting β-agonists (LABA) and long-acting muscarinic antagonists (LAMA) are mainly used as pharmacological therapies to prevent acute exacerbations of COPD, reduce symptoms, and minimize the rate of lung function decline. As current therapies do not modify the course of the disease, developing new therapeutic strategies aiming to regenerate tissue is necessary (Wu et al., 2022).

### 7.1 Pericytes in COPD

Pericytes have a critical role in blood vessel wall stabilization, vessel dilation, and vascular perfusion within the microcirculation. Endothelial cells form tubes as well as complex vascular networks and facilitate pericyte recruitment simultaneously (Payne et al., 2019). While the pericyte regulates the capillary permeability, the defective pericyte distorts the pericyte-endothelial cell interaction resulting in a chaotic, poorly organized and dysfunctional vasculature (Pinto-Plata et al., 2007; Kato et al., 2018; Meng et al., 2021). Increasing evidence indicates that pericyte dysfunction is involved in COPD pathology, which is characterized by airway remodelling, bronchitis (inflammation), and emphysema (alveolar destruction). Various risk factors lead to COPD, including tobacco smoking, air pollution, genetic susceptibility, and abnormal early life events (Hiemstra et al., 2021). However, there are only a few studies directly investigating the role of pulmonary pericytes in COPD with limited molecular mechanisms.

It has been shown that tobacco smoke decreases pulmonary HIF-2α (hypoxia-inducible factor-2α) expression, which results in an emphysematous pathology. Based on this observation, Pasupneti et al. established an emphysematous model using endothelial *Hif-2α*-knockout mice, where they observed a reduction of hepatocyte growth factor (HGF) expression, which resulted from the loss of endothelial cells and pericytes (Pasupneti et al., 2020). In another study, Pakhomova et al. established a combined murine emphysema and metabolic impairment model induced by cigarette smoke extract (CSE) and sodium glutamate, where they observed a reduction of pericytes (CD31-/CD34-/CD146+) (Pakhomova et al., 2020).

Kress et al. evaluated the impact of benzo [a]pyrene diol epoxide (BPDE), a major genotoxic component in cigarette smoke and air pollution, on primary human endothelial cells (HUVECs), primary human SMCs and primary human pericytes (Kress et al., 2019), respectively. Both the HUVECs and human pericytes showed less cell viability and enhanced apoptosis as well as necrosis in response to BPDE. Compared with primary HUVECs, human pericytes showed only weak induction of premature senescence and much lower levels of DNA damage.

Additionally, advanced age is also a major risk factor for developing COPD. The ageing lung is characterized by increased cell adhesion and stress responses, with reduced mitochondria and cellular replication (Chow et al., 2021; Schneider et al., 2021). In a transcriptomic study using deconvolution analysis, the proportions of alveolar epithelial type 2 cells decrease with age whereas pericytes increase with age (Chow et al., 2021). However, how age-associated molecular alterations contribute to COPD pathogenesis remains largely unknown in pericytes. Blervaque et al. discovered an impaired pericyte coverage of muscle capillaries in patients of mild-to-moderate COPD (Blervaque et al., 2020); this pericyte impairment may be due to inflammation, as both IL-1β and TNF-ɑ levels are increased in the serum of COPD patients (Pinto-Plata et al., 2007; Zou et al., 2017). Furthermore, Gouzi et al. showed a higher plasma Ang2/Ang1 ratio in COPD patients and found that this ratio was inversely correlated with the pericyte coverage index (Gouzi et al., 2022).

## 8 Conclusion

Pericytes are an underappreciated cell type with important roles in lung health and disease. Based on the available evidence, pericytes play important roles in the initiation and maintenance of chronic lung diseases (Table 1). Given their location at the interface between the airways and the vasculature, pericytes are not only exquisitely sensitive to their surrounding environment but also amenable to pharmacological interventions. Further research is required to further understand the mechanisms of pericytes in various lung diseases and efforts to create standards for lung pericyte isolation, characterisation, and cell culture are needed.

**TABLE 1:** Summary of the contribution of pericytes to pulmonary diseases as well as the key compounds involved.

Disease	Contribution of pericytes	Mediators involved
Pulmonary hypertension	- Changes to pericyte-endothelial interactions	- TGF-β
	- Migration of pericytes	- CXCR7
	- Accumulation of pericytes on blood vessels	- CXCR4
Asthma	- Migration of pericytes	- TGF-β
	- Airway remodelling	- IL-4
	- Vascular leakage	- IL-13
		- CXCL12
		- Periostin
Chronic obstructive pulmonary disease	- Dysfunctional vasculature	- Ang 1/2
	- Loss of pericytes	- HIF-2α
	- Impaired pericyte coverage of capillaries	- HGF
		- IL-1β
		- TNF-ɑ
